# Chinoidine—Its Use in Malarial Fevers

**Published:** 1881-11

**Authors:** B. R. Dostor

**Affiliations:** Blakely, Ga.


### CHINOIDINE—ITS USE IN MALARIAL FEVERS.

By B. R. DOSTOR, M.D., Blakely, Ga.

Purified chinoidine is a remedial agent the merits of which, in the treatment and prevention of malarial fevers, have, I apprehend, been unjustly overlooked, if not entirely ignored, by a great many members of the medical profession in this section of the country.

I have been using it since 1860, and for the last sixteen years, very extensively. In my hands it has proven effective; yielding the most gratifying and satisfactory results in the treatment of chills and fever. I cannot now recall a single failure, with this remedy, in this class of cases, where my directions were fully and faithfully observed.

The manufacturers tell us that it is "the residual product of the mother waters" after making quinine, etc. Judging from its effects it must contain a great deal of the valuable qualities of the cinchona bark.

Commercially, it is regarded as lowest in value of any of the cinchona products; yet, medicinally, it is entitled to far greater respect than is accorded it.

I have conversed with many good practitioners whose sights were so elevated on quinine that they were ignorant of even the appearance of chinoidine.

It comes to us occasionally now, pulverized, and at an increased cost, but heretofore it has been put up in one ounce rolls, and so it is, as a rule, at present; the mass being, at all seasons, of a very convenient consistency for rolling into pills. Some specimens are softer than others, but during the coldest weather a slight degree of artificial heat may be necessary to render it maleable. In summer the pills are inclined to flatten or spread out, which may be obviated by working in equal parts of ground black pepper and licorice, a grain to the pill will be sufficient, or better, if practicable, the pills may be coated with gelatine.

I am in the habit of administering it in doses of four to six grains, commencing a sufficient time before the expected chill, and repeating it every two hours until, in adults, eighteen to twenty-four grains have been taken.

The drug seems to dissolve readily in the stomach, causing little or no gastric irritation—if premised by a cathartic, none—and it does not affect the head unpleasantly, unless the patient should be exposed to the rays of the sun, then, they tell me, it produces a sense of fullness and a dull roaring sound, not so intense, however, as the same symptoms following the administration of quinine.

In intermittent fever, after missing one paroxysm, the patient should continue to take four grains of the remedy upon retiring at night. Taken in this way it acts as a tonic and hepatic stimulant; it clears up the skin, restores the appetite and relieves that feeling of general malaise, and the aching, yawning and "stretching" that characterize ordinary intermittent attacks, and which disqualify one for the active duties of life.

When, from any cause, the pilular form is objectionable, I dissolve the chinoidine in alcohol, in the proportion of half a troy ounce of the former to one fluid ounce of the latter, flavored with such aromatic oils, previously dissolved in the alcohol, as may best suit the taste, then add simple syrup or the red elixir in quantity sufficient to make each teaspoonful represent three grains of the substance. It should be administered in the same quantity recommended for the mass, before the expected chill or when the fever is declining.

I am confident that I have prevented a great many cases of "hemorrhagic malarial fever" by the timely use of this drug, and I have broken the fever in this condition with no other antiperiodic. It is safer in a jaundiced condition of the system than quinine; it does not increase the hemorrhage or transudation of the hæmatin like quinine, and the stomach retains it better, which is quite an item in the treatment of this, the first cousin to old "yellow jack."

I do not wish to be understood as advising reliance upon it in what is known as malignant intermittent fever, or "congestive chill," but to insist upon its efficacy in ordinary intermittent and mild cases of remittent fever, hemorrhagic malarial fever and malarial cachexia. This is its proper sphere, and here it will be found most useful.

My conclusions, then, after a long and observant use of the remedy may be briefly stated as follows: It is efficient; it is safe; it is cheap. These constitute a trio of qualities which certainly ought to commend chinoidine to a more favorable and respectful consideration at the hands of the profession throughout the country than it has hitherto received.

